# A Gauss-Kuzmin Theorem for Continued Fractions Associated with Nonpositive Integer Powers of an Integer *m* ≥ 2

**DOI:** 10.1155/2014/984650

**Published:** 2014-02-23

**Authors:** Dan Lascu

**Affiliations:** Mircea cel Batran Naval Academy, 1 Fulgerului, 900218 Constanta, Romania

## Abstract

We consider a family {*τ*_*m*_ : *m* ≥ 2} of interval maps which are generalizations of the Gauss transformation. For the continued fraction expansion arising from *τ*_*m*_, we solve a Gauss-Kuzmin-type problem.

## 1. Introduction

Chan considered some continued fraction expansions related to random Fibonacci-type sequences [1, 2]. In [1], he studied the continued fraction expansions of a real number in the closed interval [0,1] whose digits are differences of consecutive nonpositive integer powers of 2 and solved the corresponding Gauss-Kuzmin-Lévy theorem. In fact, Chan has studied the transformation related to this new continued fraction expansion and the asymptotic behaviour of its distribution function. Giving a solution to the Gauss-Kuzmin-Lévy problem, he showed in [1, Theorem 1.1] that the convergence rate involved is *𝒪*(*q* ^*n*^) as *n* → *∞* with 0 < *q* < 1.

The purpose of this paper is to prove a Gauss-Kuzmin-type problem for the continued fraction expansions of real numbers in [0,1] whose digits are differences of consecutive nonpositive integer powers of an integer *m* ≥ 2. In this section, we show our motivation and main theorems.

### 1.1. Gauss' Problem and Its Progress

One of the first and still one of the most important results in the metrical theory of continued fractions is the so-called Gauss-Kuzmin theorem. Any irrational 0 < *x* < 1 can be written as the infinite regular continued fraction

(1) $$\begin{array}{c} x=\frac{1}{a_{1}+\frac{1}{a_{2}+\frac{1}{a_{3}+\ddots }}}≔\left[a_{1},a_{2},a_{3},\ldots \right], \end{array}$$

where *a*_*n*_ ∈ *ℕ*_+_≔{1,2, 3,…}.

Roughly speaking, the metrical theory (or, as called by Khintchine, the measure theory) of continued fraction expansions is about properties of the sequence (*a*_*n*_)_*n*∈*ℕ*_+__. It started on October 25, 1800, with a note by Gauss in his mathematical diary (entry 113) [3]. Define the *regular continued fraction transformation τ* on the closed interval *I*≔[0,1] by

(2) $$\begin{array}{c} \tau \left(x\right)=\{\begin{array}{cc} \frac{1}{x}-\left\lfloor \frac{1}{x}\right\rfloor & \text{if}\;x\neq 0,\\ 0 & \text{if}\;x=0, \end{array} \end{array}$$

where ⌊·⌋ denotes the floor (or entire) function. In modern notation, Gauss wrote that “for very simple argument” we have

(3) $$\begin{array}{c} \underset{n\to \infty }{\lim}\lambda \left(\tau^{n}\leq x\right)=\frac{\log\left(1+x\right)}{\log2}\;\left(x\in I\right), \end{array}$$

where *λ* denotes the Lebesgue measure on *I* and *τ* ^*n*^ is the *n*th iterate of *τ*.

Nobody knows how Gauss found (3), and his achievement is even more remarkable if we realize that modern probability theory and ergodic theory had started almost a century later. In general, finding the invariant measure is a difficult task.

Twelve years later, in a letter dated January 30, 1812, Gauss wrote to Laplace that he did not succeed in solving satisfactorily “a curious problem” and that his efforts “were unfruitful.” In modern notation, this problem is to estimate the error

(4) $$\begin{array}{c} e_{n}\left(x\right)≔\lambda \left(\tau^{-n}\left[0,x\right]\right)-\frac{\log\left(1+x\right)}{\log2}\;\left(n\geq 1,\;\;x\in I\right). \end{array}$$

This has been called *Gauss' Problem*. It received a first solution more than a century later, when Kuzmin [4] showed in 1928 that

(5) $$\begin{array}{c} e_{n}\left(x\right)=𝒪\left(q^{\sqrt{n}}\right) \end{array}$$

as *n* → *∞*, uniformly in *x* with some (unspecified) 0 < *q* < 1. This has been called the *Gauss-Kuzmin theorem* or the *Kuzmin theorem*.

One year later, using a different method, Lévy [5] improved Kuzmin's result by showing that

(6) $$\begin{array}{c} \left|e_{n}\left(x\right)\right|\leq q^{n}, \end{array}$$

*n* ∈ *ℕ*_+_, 0 ≤ *x* < 1, with $q=3.5-2\sqrt{2}=0.67157\ldots$. The Gauss-Kuzmin-Lévy theorem is the first basic result in the rich metrical theory of continued fractions.

By such a development, generalizations of these problems for nonregular continued fractions are also called the *Gauss-Kuzmin problems*.

### 1.2. Chan's Continued Fraction Expansions

In this paper, we consider a generalization of the Gauss transformation and prove an analogous result. Especially, we will solve its Gauss-Kuzmin problem in Theorem 3.

This transformation was studied in detail by Chan in [2] and Lascu in [6].

Fix an integer *m* ≥ 2. In [2], Chan shows that any *x* ∈ [0,1) can be written as the form

(7) $$\begin{array}{c} x=\frac{m^{-a_{1}}}{1+\frac{\left(m-1\right)m^{-a_{2}}}{1+\frac{\left(m-1\right)m^{-a_{3}}}{1+\ddots }}}≔{\left[a_{1},a_{2},a_{3},\ldots \right]}_{m}, \end{array}$$

where *a*_*n*_'s are nonnegative integers. Such *a*_*n*_'s are also called *incomplete quotients (or continued fraction digits)* of *x* with respect to the expansion in (7) in this paper.

This continued fraction is treated as the following dynamical systems.

**Definition 1**

Fix an integer *m* ≥ 2.

(i) The measure-theoretical dynamical system (*I*, *ℬ*_*I*_, *τ*_*m*_) is defined as follows: *I*≔[0,1], where *ℬ*_*I*_ denotes the *σ*-algebra of all Borel subsets of *I* and *τ*_*m*_ is the transformation
  (8) τm:I⟶I,τm(x)≔{1m−1(1mi  x−1)if  x∈Ii,0if  x=0,
  where *I*_*i*_≔{*x* ∈ *I* : *m* ^−(*i*+1)^ < *x* ≤ *m* ^−*i*^} for *i* ∈ *ℕ*≔{0,1, 2,…}.
(ii) In addition to (i), one writes (*I*, *ℬ*_*I*_, *γ*_*m*_, *τ*_*m*_) as (*I*, *ℬ*_*I*_, *τ*_*m*_) with the following probability measure *γ*_*m*_ on (*I*, *ℬ*_*I*_):
  (9) γm(A)≔km∫Adx{(m−1)x+1}  {(m−1)x+m}(A∈ℬI),
  where
  (10) $$\begin{array}{c} k_{m}≔\frac{{\left(m-1\right)}^{2}}{\log\left\{m^{2}/\left(2m-1\right)\right\}}. \end{array}$$

Define the *quantized index map* *η*_*m*_ : *I* → *ℕ* by

(11) $$\begin{array}{c} \eta_{m}\left(x\right)≔\{\begin{array}{cc} \left\lfloor -\log_{m}x\right\rfloor & \text{if}\;x\neq 0,\\ \infty & \text{if}\;x=0. \end{array} \end{array}$$

By definition, *η*_*m*_(*m* ^−*α*^) = ⌊*α*⌋. By using *τ*_*m*_ and *η*_*m*_, the sequence (*a*_*n*_)_*n*∈*ℕ*_+__ in (7) is obtained as follows:

(12) $$\begin{array}{c} a_{n}=\eta_{m}\left(\tau_{m}^{n-1}\left(x\right)\right)\;\left(n\geq 1\right) \end{array}$$

with *τ*_*m*_^0^(*x*) = *x*. In this way, *τ*_*m*_ gives the algorithm of Chan's continued fraction expansion (7).

**Proposition 2**

Let (*I*, *ℬ*_*I*_, *γ*_*m*_, *τ*_*m*_) be as in Definition 1(ii).

- (*I*, *ℬ*_*I*_, *γ*_*m*_, *τ*_*m*_) is ergodic.
- The measure *γ*_*m*_ is invariant under *τ*_*m*_; that is, *γ*_*m*_(*A*) = *γ*_*m*_(*τ*_*m*_^−1^(*A*)) for any *A* ∈ *ℬ*_*I*_.

**Proof**

See [2, 6].

By Proposition 2(ii), (*I*, *ℬ*_*I*_, *γ*_*m*_, *τ*_*m*_) is a “dynamical system” in the sense of [7, Definition 3.1.3].

### 1.3. Known Results and Applications

For Chan's continued fraction expansions, we show known results and their applications in this subsection.

#### 1.3.1. Known Results for *m* = 2 Case

For (*I*, *ℬ*_*I*_, *γ*_*m*_, *τ*_*m*_) in Definition 1(ii), assume *m* = 2; that is, we consider only (*I*, *ℬ*_*I*_, *γ*_2_, *τ*_2_) in here.

In [8], Chan proved a Gauss-Kuzmin-Lévy theorem for the transformation *τ*_2_. He showed that the convergence rate of the *n*th distribution function of *τ*_2_ to its limit is *𝒪*(*q* ^*n*^) as *n* → *∞* with *q* ≤ 0.880555 uniformly in *x*.

In [9, 10], Sebe investigated the Perron-Frobenius operator of *τ*_2_ by replacing a probability measure of the measurable space (*I*, *ℬ*_*I*_). Especially, Sebe studied the Perron-Frobenius operator *J* of (*I*, *ℬ*_*I*_, *γ*_2_, *τ*_2_); that is, *J* is a unique operator on *L* ^1^(*I*, *γ*_2_) satisfying

(13) ∫A{Jf}(x)dγ2(x)=∫τ2−1(A)f(x)dγ2(x)(A∈ℬI,f∈L1(I,γ2)).

The asymptotic behavior of *J* was shown by using well-known general results [11, 12]. By a Wirsing-type approach [13], Sebe obtained a better estimate of the convergence rate involved [9]. In fact, its upper and lower bounds of the convergence rate were obtained as *𝒪*(*w* ^*n*^) and *𝒪*(*v* ^*n*^), respectively, when *n* → *∞*, with *w* < 0.209364308 and *v* > 0.206968896 ([9, Theorem 4.3]). They provide a near-optimal solution to the Gauss-Kuzmin-Lévy problem.

Furthermore, by restricting the Perron-Frobenius operator to the Banach space of functions *f* : *I* → *ℂ* of bounded variation, Iosifescu and Sebe [14] proved that the exact optimal convergence rate of *γ*_*a*_  (*s*_*n*_^*a*^ ≤ *x*) to *γ*_2_([0, *x*]) is *𝒪*(*g* ^2*n*^) as *n* → *∞* uniformly in *x*. Here *g* is the inverse of the golden ratio; that is, we have

(14) $$\begin{array}{c} g=\frac{2}{\sqrt{5}+1},\;g^{2}+g=1,\;g^{2}=\frac{3-\sqrt{5}}{2}=0.38196\ldots . \end{array}$$

For *a* ≥ 0, define the sequence (*s*_*n*,*a*_)_*n*∈*ℕ*_ recursively by *s*_*n*,*a*_≔2^−*a*_*n*_^/(1 + *s*_*n*−1,*a*_), *n* ∈ *ℕ*_+_, with *s*_0,*a*_≔*a*. Then it is an *I* ∪ {*a*}-valued Markov chain on (*I*, *ℬ*_*I*_, *γ*_*a*_) where *γ*_*a*_ is the probability measure on (*I*, *ℬ*_*I*_) defined as the following distribution function:

(15) $$\begin{array}{c} \gamma_{a}\left(\left[0,x\right]\right)=:\frac{\left(a+2\right)x}{x+a+1}\;\left(x\in I,\;\;a\geq 0\right). \end{array}$$

For *J* in (13), let *J*′ denote its restriction on *L* ^*∞*^(*I*) ⊂ *L* ^1^(*I*, *γ*_2_). From [12, Proposition 2.1.10], we see that *J*′ is the transition operator of the Markov chain (*s*_*n*,*a*_)_*n*∈*ℕ*_+__ on (*I*, *ℬ*_*I*_, *γ*_*a*_) for any *a* ≥ 0.

#### 1.3.2. Known Results for *m* ≥ 3 Case

For (*I*, *ℬ*_*I*_, *γ*_*m*_, *τ*_*m*_) in Definition 1(ii), recall the main results in [6, 15].

In [6], Lascu proved a Gauss-Kuzmin theorem for the transformation *τ*_*m*_. In order to solve the problem, he applied the theory of random systems with complete connections (RSCC) by Iosifescu and Grigorescu [11]. We remind that a random system with complete connections is a quadruple

(16) $$\begin{array}{c} \left\{\left(I,{ℬ}_{I}\right),\left({\mathbb{N}}_{+},𝒫\left({\mathbb{N}}_{+}\right)\right),u,P\right\}, \end{array}$$

where *u* : *I* × *ℕ* → *I*,

(17) $$\begin{array}{c} u\left(x,i\right)=u_{m,i}\left(x\right)=\frac{m^{-i}}{\left(m-1\right)x+1}\;\left(x\in I\right), \end{array}$$

and *P* is the transition probability function from (*I*, *ℬ*_*I*_) to (*ℕ*, *𝒫*(*ℕ*)) given by

(18) P(x,i)=Pm,i(x)=(m−1)m−(i+1)(x+1)(x+m)(x+(m−1)m−i+1)(x+(m−1)m−(i+1)+1).

Also, the associated Markov operator of RSCC (16) is denoted by *U*_*m*_ and has the transition probability function

(19) $$\begin{array}{c} Q_{m}\left(x,A\right)=\sum_{i\in W_{m}\left(x,A\right)}P_{m,i}\left(x\right)\;\left(x\in I,A\in {ℬ}_{I}\right), \end{array}$$

where *W*_*m*_(*x*, *A*) = {*i* ∈ *ℕ* : *u*_*m*,*i*_(*x*) ∈ *A*}.

Using the asymptotic and ergodic properties of operators associated with RSCC (16), that is, the ergodicity of RSCC, he obtained a convergence rate result for the Gauss-Kuzmin-type problem.

For more details about using RSCC in solving the Gauss-Kuzmin-Lévy-type theorems, see [11, 16–20].

By a Wirsing-type approach [13] to the Perron-Frobenius operator of the associated transformation under its invariant measure, Sebe [15] studied the optimality of the convergence rate. Actually, Sebe obtained upper and lower bounds of the convergence rate which provide a near-optimal solution to the Gauss-Kuzmin-Lévy problem. In the case *m* = 3, the upper and lower bounds of the convergence rate were obtained as *O*(*w*_3_^*n*^) and *O*(*v*_3_^*n*^), respectively, when *n* → *∞*, with *v*_3_ > 0.262765464 and *w*_3_ < 0.264687208.

#### 1.3.3. Application to the Asymptotic Growth Rate of a Fibonacci-Type Sequence

We explain an application of *τ*_*m*_ to a Fibonacci-type sequence here. As it is known, the *Fibonacci sequence *(*F*_*n*_) is recursively defined as follows:

(20) $$\begin{array}{c} F_{0}=F_{1}=1,\;\;F_{n}=F_{n-1}+F_{n-2}\;\left(n\geq 2\right). \end{array}$$

Equivalently, (*F*_*n*_) is also defined by *Binet's formula* $F_{n}=(G^{n+1}-G^{-(n+1)})/\sqrt{5}$ for *n* ≥ 0 where $G:=(1+\sqrt{5})/2$ is the golden ratio. By this formula, the asymptotic growth rate of (*F*_*n*_) is obtained as follows:

(21) $$\begin{array}{c} \underset{n\to \infty }{\lim}\frac{1}{n}\log F_{n}=\log\frac{1+\sqrt{5}}{2}=0.4812\ldots . \end{array}$$

A *random Fibonacci sequence *(*f*_*n*_) is defined as (with fixed *f*_1_ and *f*_2_)

(22) $$\begin{array}{c} f_{n}=\alpha \left(n\right)f_{n-1}+\beta \left(n\right)f_{n-2}, \end{array}$$

where *α*(*n*) and *β*(*n*) are random coefficients. For such (*f*_*n*_), the quest for its asymptotic growth rate is more difficult. We show two examples of random Fibonacci sequences as follows.

(i) Define the random Fibonacci sequence (*f*_*n*_) as
  (23) $$\begin{array}{c} f_{1}=f_{2}=1,\;\;f_{n}=\pm f_{n-1}\pm f_{n-2}, \end{array}$$
  where the signs in (23) are chosen independently and with equal probabilities. Recently, Viswanath [21] has proved that its asymptotic growth rate is given as
  (24) $$\begin{array}{c} \underset{n\to \infty }{\lim}\frac{1}{n}\log\left|f_{n}\right|=\log\left(1.13198824\ldots \right)=0.12397559\ldots \end{array}$$
  with probability 1.
(ii) Fix an integer *m* ≥ 2. Define the random Fibonacci sequence (*f*_*n*_) as
  (25) f−1=0,  f0=1,  a0=0,  fn=manfn−1+(m−1)man−1fn−2,
  where *a*_*n*_'s are as in (7). By using the ergodicity of (*I*, *ℬ*_*I*_, *γ*_*m*_, *τ*_*m*_) (Proposition 2(i)), Chan proved that its asymptotic growth rate is given as follows [2]:
  (26) ηm≔lim⁡n→∞1nlog⁡fn=km∫01−log⁡t{(m−1)t+1}{(m−1)t+m}  dt≤km3m−12m(2m−1),
  where *k*_*m*_ is as in (10).

#### 1.3.4. A Khintchine-Type Result and Entropy

In probabilistic number theory, statistical limit theorems are established in problems involving “almost independent” random variables. The nonnegative integers *a*_*n*_, *n* ∈ *ℕ*_+_, define random variables on the measure space (*I*, *ℬ*_*I*_, **P**), where **P** is a probability measure on *I*.

Continued fraction expansions of almost all irrational numbers are not periodic. Nevertheless, we readily reproduce another famous probabilistic result. It is the asymptotic value *χ*_*m*_ of the geometric mean of *m* ^*a*_1_^, *m* ^*a*_2_^,…, *m* ^*a*_*n*_^; that is,

(27) $$\begin{array}{c} \chi_{m}≔\underset{n\to \infty }{\lim}\log{\left(m^{a_{1}+a_{2}+\cdots +a_{n}}\right)}^{1/n}, \end{array}$$

where *a*_*n*_'s are given in (12). This is a Khintchine-type result and we obtain

(28) χm=(log⁡⁡m)∫01a1(x)ρm(x)dx=kmlog⁡⁡m(m−1)2 ×∑n=0∞log⁡⁡(1+(m−1)3mn+2+2(m−1)m+((m−1)2/mn))n

for almost all real numbers *x* = [*a*_1_(*x*), *a*_2_(*x*), *a*_3_(*x*),…]_*m*_ ∈ (0,1). As it can be seen, *χ*_*m*_ is a constant independent of the value of *x*.

As it is well known, entropy is an important concept of information in physics, chemistry, and information theory [22]. The connection between entropy and the transmission of information was first studied by Shannon in [23]. The entropy can be seen as a measure of randomness of the system or the average information acquired under a single application of the underlying map. Entropy also plays an important role in ergodic theory. Thus in 1958 Kolmogorov [24] imported Shannon's probabilistic notion of entropy into the theory of dynamical systems and showed how entropy can be used to tell whether two dynamical systems are nonconjugate. Like Birkhoff's ergodic theorem [22] the entropy is a fundamental result in ergodic theory. For a measure preserving transformation, its entropy is often defined by using partitions, but in 1964 Rohlin [25] showed that the entropy of a *μ*-measure preserving operator *T* : [*a*, *b*]→[*a*, *b*] is given by the beautiful formula

(29) $$\begin{array}{c} h\left(T\right)≔\int_{a}^{b}\log\left|T^{\prime }\left(x\right)\right|d\mu \left(x\right). \end{array}$$

From Rohlin's formula it follows that the entropy of the operator *τ*_*m*_ in (8) on the unit interval with respect to the measure *γ*_*m*_ in (9) is given by

(30) h(τm)=∫01log⁡⁡|τm′(x)|ρm(x)dx=∫01log⁡⁡(m−a1(x)(m−1)x2)ρm(x)dx=2ηm−χm−log⁡⁡(m−1),

where *a*_1_, *η*_*m*_, and *χ*_*m*_ are given in (12), (26), and (27), respectively.

### 1.4. Main Theorem and Its Consequences

#### 1.4.1. Main Theorem

We show our main theorems in this subsection. Fix an integer *m* ≥ 2. Let *k*_*m*_ be as in (10) and let (*I*, *ℬ*_*I*_) be as in Section 1.2. If *x* has the expansion in (7) and *τ*_*m*_ is as in (8), then the question about the asymptotic distribution of *τ*_*m*_^*n*^ appears. If we know this, then the corresponding probability that *a*_*n*+1_ = *i* is simply written as prob(*m* ^−(*i*+1)^ < *τ*_*m*_^*n*^ < *m* ^−*i*^). We will show that the event *τ*_*m*_^*n*^ ≤ *x* has the following asymptotic probability:

(31) $$\begin{array}{c} \omega_{m}\left(x\right)=\frac{k_{m}}{{\left(m-1\right)}^{2}}\log\frac{m\left(\left(m-1\right)x+1\right)}{\left(m-1\right)x+m}\;\left(x\in I\right). \end{array}$$

This result allows us to say that the probability density function

(32) $$\begin{array}{c} \rho_{m}\left(x\right)=\frac{k_{m}}{\left(\left(m-1\right)x+1\right)\left(\left(m-1\right)x+m\right)} \end{array}$$

is invariant under *τ*_*m*_: if a random variable *X* in the unit interval has the density *ρ*_*m*_, and then so does *τ*_*m*_. The reason for this invariance is that, for 0 ≤ *x* < *x* + *h* ≤ 1, *τ*_*m*_ lies between *x* and *x* + *h* if and only if there exists *i* ≥ 1, so that *X* lies between 1/(*x* + *i* + *h*) and 1/(*x* + *i*). Thus

(33) $$\begin{array}{c} \text{prob}\left(x\leq \tau_{m}\leq x+h\right)=\sum_{i\in {\mathbb{N}}_{+}}\text{prob}\left(\frac{1}{x+i+h}\leq X\leq \frac{1}{x+i}\right). \end{array}$$

Taking the limit as *h* → *∞* gives that, for an arbitrary probability density function *f* for *X*, the corresponding density *Gf* for *τ*_*m*_ is given a.e. in *I* by the equation

(34) $$\begin{array}{c} Gf\left(x\right)=\sum_{i\in \mathbb{N}}\frac{\left(m-1\right)m^{-i}}{{\left(\left(m-1\right)x+1\right)}^{2}}f\left(\frac{m^{-i}}{\left(m-1\right)x+1}\right). \end{array}$$

Clearly, the operator *G* : *L* ^1^ → *L* ^1^ admits the density function *ρ*_*m*_ as an eigenfunction corresponding to the eigenvalue 1; that is, *Gρ*_*m*_ = *ρ*_*m*_. Here *L* ^1^ denotes the Banach space of all complex functions *f* : *I* → *ℂ* for which ∫_*I*_|*f*|*dλ* < *∞*.

The only eigenvalue of modulus 1 of *G* is 1 and this eigenvalue is simple.

From another perspective, the operator *τ*_*m*_ is an ergodic operator on the unit interval [2], *ρ*_*m*_ is the density of the invariant measure, and *G* is called *transfer operator* for *τ*_*m*_ [6]. The transfer operator *G* has the same analytical expression as the Perron-Frobenius operator of *τ*_*m*_ under the Lebesgue measure [6].

Our main result is the following theorem.

**Theorem 3 (the Gauss-Kuzmin theorem)**

Let *τ*_*m*_ and *ω*_*m*_ be as in (8) and (31), respectively. When a nonatomic probability measure *μ* on (*I*, *ℬ*_*I*_) is given, define functions *F*_*m*,*n*_ (*n* ≥ 0) on *I* by

(35) $$\begin{array}{c} F_{m,0}\left(x\right)≔\mu \left(\left[0,x\right]\right),\\ F_{m,n}\left(x\right)≔\mu \left(\tau_{m}^{n}\leq x\right)\;\left(n\geq 1\right) \end{array}$$

for *x* ∈ *I*. Then there exists a constant 0 < *q*_*m*_ < 1 such that *F*_*m*,*n*_ is written as

(36) $$\begin{array}{c} F_{m,n}\left(x\right)=\omega_{m}\left(x\right)+𝒪\left(q_{m}^{n}\right). \end{array}$$

**Remark 4**

(i) From (36), we see that

(37) $$\begin{array}{c} \underset{n\to \infty }{\lim}F_{m,n}\left(x\right)=\gamma_{m}\left(\left[0,x\right]\right), \end{array}$$

where *γ*_*m*_ is the measure defined in (9). In fact, the Gauss-Kuzmin theorem estimates the error

(38) em,n(x)=em,n(x,μ)=μ(τmn≤x)−γm([0,x])(x∈I).

(ii) The solution of this problem implies that (*a*_*n*_)_*n*∈*ℕ*_ in (12) is exponentially *ψ*-mixing under *γ*_*m*_ (and under many other probability measures including *λ*) [11, 12]; that is,

(39) |γm(A1∩A2)−γm(A1)γm(A2)|≤Cqnγm(A1)γm(A2)(n∈ℕ+)

for any *A*_1_ ∈ *σ*(*a*_1_,…, *a*_*k*_) (the *σ*-algebra generated by the random variables *a*_1_,…, *a*_*k*_), *A*_2_ ∈ *σ*(*a*_*n*+*k*_, *a*_*n*+*k*+1_,…), and *k* ∈ *ℕ*_+_, with suitable positive constants *q* < 1 and *C*.

In turn, *ψ*-mixing implies lots of limit theorems in both classical and functional versions. To form an idea of the results to be expected it is sufficient to look at the corresponding results for the regular continued fraction expansions [12].

(iii) In (37) we emphasized the probabilistic nature of Gauss' result. Khintchine [26] and Doeblin [27] found new probabilistic results on the regular continued fraction transformation. These types of results were established also for the transformation *τ*_*m*_ [2, 6]. These results establish, among other properties, that the map *τ*_*m*_ is ergodic (Proposition 2(i)). Kuzmin's theorem may then be rephrased by saying that the convergence encountered in the mixing process (the “approach to equilibrium”) is in fact exponential. If we define the linear operator Π_1_ by

(40) $$\begin{array}{c} \Pi_{1}f\left(x\right)=\rho_{m}\left(x\right)\int_{I}fd\lambda \;\left(f\in L^{1},x\in I\right), \end{array}$$

then there exists 0 < *q*_*m*_ < 1 such that

(41) $$\begin{array}{c} {||G^{n}-\Pi_{1}||}_{v}\leq 𝒪\left(q_{m}^{n}\right)\;\left(n⟶\infty \right). \end{array}$$

The norm ||·||_*v*_ is defined by ||*f*||_*v*_ = ||*f*|| + *“*total  variation  of  *f”* [12].

**Problem 5**

(i) Solve the Gauss-Kuzmin-Lévy problem of *τ*_*m*_ for *m* ≥ 3. For example, study the optimality of the convergence rate. Use the same strategy as in [14].

(ii) It is known that the Riemann zeta function is written by using a kind of Mellin transformation of the Gauss transformation *τ* in (2) as follows [28]:

(42) $$\begin{array}{c} \zeta \left(s\right)=\frac{1}{s-1}-s\int_{0}^{1}\tau \left(x\right)x^{s-1}\;dx\;\left(0<ℜ\left(s\right)<1\right). \end{array}$$

This is derived by using the Euler-Maclaurin summation formula ([29, page 14]) and the definition of *τ*. Then, by replacing *τ* with *τ*_*m*_ in (8), can we regard

(43) $$\begin{array}{c} Z_{m}\left(s\right)≔\frac{1}{s-1}-s\int_{0}^{1}\tau_{m}\left(x\right)x^{s-1}\;dx \end{array}$$

as a new zeta function?

The rest of the paper is organised as follows. In Section 2, we prove Theorem 3. In Section 2.1, we give the necessary results used to prove the Gauss-Kuzmin theorem for the continued fractions presented in Section 1. The essential argument of the proof is the Gauss-Kuzmin-type equation. We will also give some results concerning the behavior of the derivative of {*F*_*m*,*n*_} in (35) which will allow us to complete the proof of Theorem 3 in Section 2.2.

## 2. Proof of Theorem 3

In this section, we will prove Theorem 3 applying the method of Rockett and Szüsz [30]. Fix an integer *m* ≥ 2.

### 2.1. Necessary Lemmas

In this subsection, we show some lemmas. First, we show that {*F*_*m*,*n*_} in (35) satisfy a Gauss-Kuzmin-type equation.

**Lemma 6**

For functions {*F*_*m*,*n*_} in (35), the following Gauss-Kuzmin-type equation holds:

(44) $$\begin{array}{c} F_{m,n+1}\left(x\right)=\sum_{i\in \mathbb{N}}\left\{F_{m,n}\left(\alpha^{i}\right)-F_{m,n}\left(\frac{\alpha^{i}}{1+\left(m-1\right)x}\right)\right\} \end{array}$$

for *x* ∈ [0,1] and *n* ∈ *ℕ* where *α* = 1/*m*.

**Proof**

Let *I*≔[0,1], *I*_*m*,*n*_ = {*x* ∈ *I* : *τ*_*m*_^*n*^(*x*) ≤ *x*}, and *I*_*m*,*n*,*i*_ = {*x* ∈ *I*_*m*,*n*_ :   *α* ^*i*^/(1 + (*m* − 1)*x*) < *τ*_*m*_^*n*^(*x*) < *α* ^*i*^}.

From (8) and (12), we see that

(45) $$\begin{array}{c} \tau_{m}^{n}\left(x\right)=\frac{m^{-a_{n+1}(x)}}{1+\left(m-1\right)\tau_{m}^{n+1}\left(x\right)}\;\left(n\in {\mathbb{N}}_{+}\right). \end{array}$$

From the definition of *I*_*m*,*n*,*i*_ and (45) it follows that, for any *n* ∈ *ℕ*, *I*_*m*,*n*+1_ = ⋃_*i*∈*ℕ*_ *I*_*m*,*n*,*i*_. From this and using the *σ*-additivity of *μ*, we have

(46) $$\begin{array}{c} \mu \left(I_{m,n+1}\right)=\mu \left(\underset{i\in \mathbb{N}}{⋃}I_{m,n,i}\right)=\sum_{i\in \mathbb{N}}\mu \left(I_{m,n,i}\right). \end{array}$$

Then (44) holds because *F*_*m*,*n*+1_(*x*) = *μ*(*I*_*m*,*n*+1_) and

(47) $$\begin{array}{c} \mu \left(I_{m,n,i}\right)=F_{m,n}\left(\alpha^{i}\right)-F_{m,n}\left(\frac{\alpha^{i}}{1+\left(m-1\right)x}\right). \end{array}$$

**Remark 7**

Assume that, for some *p* ∈ *ℕ*, the derivative *F*_*p*_′ exists everywhere in *I* and is bounded. Then it is easy to see by induction that *F*_*m*,*p*+*n*_′ exists and is bounded for all *n* ∈ *ℕ*_+_. This allows us to differentiate (44) term by term, obtaining

(48) $$\begin{array}{c} F_{m,n+1}^{\prime }\left(x\right)=\sum_{i\in \mathbb{N}}\frac{\left(m-1\right)\alpha^{i}}{{\left(1+\left(m-1\right)x\right)}^{2}}F_{m,n}^{\prime }\left(\frac{\alpha^{i}}{1+\left(m-1\right)x}\right). \end{array}$$

We introduce functions {*f*_*m*,*n*_} as follows:

(49) fm,n(x)≔(1+(m−1)x)(m+(m−1)x)Fm,n′(x)(x∈I,n∈ℕ).

Then (48) is

(50) $$\begin{array}{c} f_{m,n+1}\left(x\right)=\sum_{i\in \mathbb{N}}P_{m}^{i}\left(\left(m-1\right)x\right)f_{m,n}\left(\frac{\alpha^{i}}{1+\left(m-1\right)x}\right), \end{array}$$

where *P*_*m*_^*i*^(*x*) is given in (18).

For *i* ∈ *ℕ*, define *δ*_*i*_ and *β*_*m*_^*i*^(*x*) by

(51) $$\begin{array}{c} \delta_{i}≔\alpha^{i}-\alpha^{2i},\\ \beta_{m}^{i}\left(x\right)≔\frac{\left(m-1\right)\delta_{i}}{{\left(\left(m-1\right)x+\left(m-1\right)\alpha^{i}+1\right)}^{2}}\;\left(x\in I\right). \end{array}$$

Then we get

(52) Pmi((m−1)x)=(m−1)[αi+1+δi(m−1)x+(m−1)αi+1−δi+1(m−1)x+(m−1)αi+1+1].

**Lemma 8**

For {*f*_*m*,*n*_} in (49), define *M*_*n*_≔max⁡_*x*∈*I*_ | *f*_*m*,*n*_′(*x*)|. Then

(53) $$\begin{array}{c} M_{n+1}\leq q_{m}\cdot M_{n}, \end{array}$$

where

(54) $$\begin{array}{c} q_{m}≔{\left(m-1\right)}^{2}\left(m^{2}+1\right)\sum_{i\in \mathbb{N}}\frac{1}{{\left(m^{i+1}+m-1\right)}^{2}}. \end{array}$$

**Proof**

We have

(55) $$\begin{array}{c} {\left(P_{m}^{i}\left(\left(m-1\right)x\right)\right)}^{\prime }=\left(m-1\right)\left(\beta_{m}^{i+1}\left(x\right)-\beta_{m}^{i}\left(x\right)\right). \end{array}$$

Now from (50) and by calculus, we have

(56) fm,n+1′(x)=(m−1)2∑i∈ℕAifm,n′(θi)−(m−1)∑i∈ℕBifm,n′(umi(x)),

where

(57) $$\begin{array}{c} A_{i}≔u_{m}^{i+1}\left(x\right)\beta_{m}^{i+1}\left(x\right),\\ B_{i}≔P_{m}^{i}\left(\left(m-1\right)x\right)\frac{\alpha^{i}}{{\left(\left(m-1\right)x+1\right)}^{2}},\\ u_{m}^{i+1}\left(x\right)<\theta_{i}<u_{m}^{i}\left(x\right). \end{array}$$

Now (56) implies

(58) $$\begin{array}{c} M_{n+1}\leq M_{n}\cdot \underset{x\in I}{\max}\left|{\left(m-1\right)}^{2}\sum_{i\in \mathbb{N}}A_{i}+\left(m-1\right)\sum_{i\in \mathbb{N}}B_{i}\right|. \end{array}$$

We now must calculate the maximum value of the sums in this expression.

First, we note that

(59) $$\begin{array}{c} A_{i}\leq \frac{\alpha^{2i+2}}{{\left(\left(m-1\right)\alpha^{i+1}+1\right)}^{2}}, \end{array}$$

where we use *δ*_*i*_ = *α* ^*i*^ − *α* ^2*i*^ and 0 ≤ *x* ≤ 1.

Next, observe that the function

(60) $$\begin{array}{c} h_{m}\left(x\right)≔\frac{\alpha^{i}P_{m}^{i}\left(\left(m-1\right)x\right)}{{\left(\left(m-1\right)x+1\right)}^{2}} \end{array}$$

is decreasing for *x* ∈ *I* and *i* ∈ *ℕ*. Hence, *h*_*m*_(*x*) ≤ *h*_*m*_(0). This leads to

(61) $$\begin{array}{c} \frac{\alpha^{i}P_{m}^{i}\left(\left(m-1\right)x\right)}{{\left(\left(m-1\right)x+1\right)}^{2}}\leq \frac{\left(m-1\right)\alpha^{2i}}{{\left(\left(m-1\right)\alpha^{i+1}+1\right)}^{2}}. \end{array}$$

The relations (58), (59), and (61) imply (53) and (54).

### 2.2. Proof of Theorem 3

Introduce a function *R*_*m*,*n*_(*x*) such that

(62) $$\begin{array}{c} F_{m,n}\left(x\right)=\omega_{m}\left(x\right)+R_{m,n}\left(\omega_{m}\left(x\right)\right). \end{array}$$

Because *F*_*m*,*n*_(0) = 0 and *F*_*m*,*n*_(1) = 1, we have *R*_*m*,*n*_(0) = *R*_*m*,*n*_(1) = 0. To prove Theorem 3, we have to show the existence of a constant 0 < *q*_*m*_ < 1 such that

(63) $$\begin{array}{c} R_{m,n}\left(x\right)=𝒪\left(q_{m}^{n}\right). \end{array}$$

If we can show that *f*_*m*,*n*_(*x*) = *k*_*m*_ + *𝒪*(*q*_*m*_^*n*^), then its integration will show (36).

To demonstrate that *f*_*m*,*n*_(*x*) has this desired form, it suffices to prove the following lemma.

**Lemma 9**

For any *x* ∈ *I* and *n* ∈ *ℕ*, there exists a constant 0 < *q*_*m*_ < 1 such that

(64) $$\begin{array}{c} f_{m,n}^{\prime }\left(x\right)=𝒪\left(q_{m}^{n}\right). \end{array}$$

**Proof**

Let *q*_*m*_ be as in (54). Using Lemma 8, to show (64) it is enough to prove that *q*_*m*_ < 1. To this end, for *i* ≥ 2, observe that

(65) $$\begin{array}{c} \frac{1}{{\left(m^{i+1}+m-1\right)}^{2}}\leq \frac{1}{m^{2}{\left(m-1\right)}^{2}\left(m^{2}+1\right)}{\left(\frac{1}{m}\right)}^{i}. \end{array}$$

Therefore

(66) qm≤(m−1)2(m2+1) ×{1(2m−1)2+1m2+m−1+1m2(m−1)2(m2+1)∑i≥2(1m)i}=(m−1)2(m2+1) ×{1(2m−1)2+1m2+m−1+1m3(m−1)3(m2+1)}≤1,

for any *m* ∈ *ℕ*, *m* ≥ 2.
